# Polishing Step Purification of High-Strength Wastewaters by Nanofiltration and Reverse Osmosis

**DOI:** 10.3390/membranes6010019

**Published:** 2016-03-10

**Authors:** Jinxiang Zhou, Brian O. Baker, Charles T. Grimsley, Scott M. Husson

**Affiliations:** Department of Chemical and Biomolecular Engineering and Animal Co-Products Research and Education Center, Clemson University, Clemson, SC 29634, USA; jinxiaz@g.clemson.edu (J.Z.); bobaker@g.clemson.edu (B.O.B.); cgrimsl@g.clemson.edu (C.T.G.)

**Keywords:** impaired water, membrane fouling, sustainability, water treatment

## Abstract

This article reports findings on the use of nanofiltration (NF) and reverse osmosis (RO) for secondary treatment of high-strength rendering facility wastewaters following an ultrafiltration step. These wastewaters present significant challenges to classical treatment technologies. Constant-pressure, direct-flow membrane filtration experiments were done to screen for flux and effluent water permeate quality of ten commercial NF and RO membranes. All membranes tested were effective in reducing total dissolved salts (TDS) and chemical oxygen demand (COD); however, only two membranes (Koch MPF-34 and Toray 70UB) gave sufficiently stable flux values to warrant longer term cross-flow filtration studies. Cross-flow flux measurements, scanning electron microscopy (SEM), X-ray dispersive spectroscopy (EDS), and attenuated total reflectance-Fourier-transform infrared spectroscopy (ATR-FTIR) indicated that both membranes were eventually fouled by organic and inorganic foulants; however, the Toray 70UB RO membrane yielded a capacity of 1600 L/m^2^ prior to cleaning. A preliminary economic analysis compared the estimated costs of energy and consumables for a dual-stage UF/RO membrane process and dissolved air floatation (DAF) and found membrane process costs could be less than about 40% of the current DAF process.

## 1. Introduction

**Figure 1:**
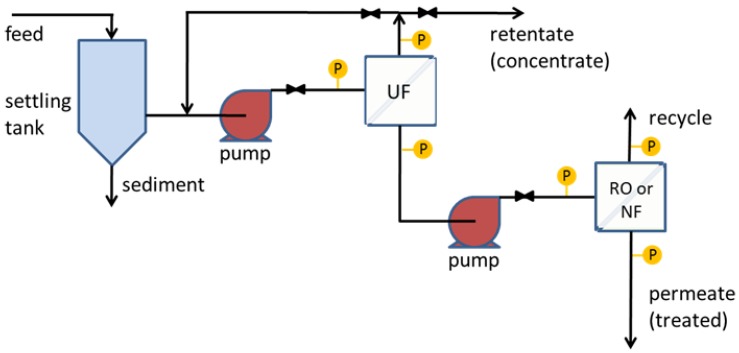
Two-stage membrane treatment process for rendering facility wastewater. The first stage uses membrane UF. This paper provides performance and economic data for the second “polishing” stage using RO or NF. P represents a pressure gauge.

The rendering industry recycles perishable materials from livestock, meat/poultry processing, food processing, and supermarkets and restaurants into valuable products. However, it also generates large volumes of high-strength wastewaters. Previously, we highlighted the advantages of membrane processes over other wastewater treatment technologies such as coagulation, flocculation, air flotation and gravity separation that are used to treat high-strength wastewaters such as those in the rendering industry [1,2]. Whereas dissolved air flotation (DAF) typically requires the use of chemicals or polymer additives to adjust the pH and improve flocculation of the solids for improved removal efficiency [3], membranes provide a positive barrier for rejection of solids that does not require such additives. Results of our work with ultrafiltration (UF) membranes [1,2] showed that it is effective in achieving significant reduction in turbidity, chemical oxygen demand (COD), and total dissolved solids (TDS) at high flux. Ultrafiltration membranes, however, are not designed for removal of low molecular weight compounds and salts that contribute to COD and TDS. Rather, they provide an initial purification step that can be followed by a polishing step such as nanofiltration (NF) or reverse osmosis (RO) to recover clean water for direct discharge or beneficial use. The use of such membrane cascades is common, and seawater desalination is one example where UF followed by RO is used in practice. In this contribution, we evaluate the use of commercial NF and RO membranes following UF in an additive-free, membrane-based wastewater treatment process for high-strength wastewaters (see Figure 1).

NF membranes are effective in removing divalent salts and organic molecules with high enough molecular weights. Compared to RO membranes, NF membranes have lower rejection of monovalent salts. Typical pore sizes of NF membranes are around 1 nm, and molecular weight cut-offs (MWCOs) are about 300–500 Da [4]. RO membranes are characterized by MWCO of about 100 Da [5]. Previous studies reported in the literature [6,7,8] have successfully applied RO and NF membranes to treat different types of high strength wastewaters, including those from the chemical industry, food industry, and agricultural wastewaters, among others. Treatment of rendering wastewater using RO or NF membranes has not been reported.

The objectives of this study were to determine the performance of commercial RO and NF membranes for secondary treatment of rendering facility wastewater following an ultrafiltration step, to characterize the membrane surfaces pre- and post-filtration to assess fouling, and to perform a preliminary cost analysis for operating a dual-stage UF/NF or UF/RO membrane separation process. Direct- and cross-flow membrane filtration experiments using wastewater provided by a rendering facility were carried out, and membrane performance was evaluated by measuring productivity (*i.e.*, the volumetric filtrate flux), capacity (*i.e.*, the total volume processed per unit membrane area before the membrane must be cleaned), and effluent water quality (COD, turbidity, TDS). Membrane fouling was detected using scanning electron microscopy (SEM), energy-dispersive X-ray spectroscopy (EDS) and attenuated total reflection Fourier-transform infrared spectroscopy (ATR-FTIR). The cost analysis compared the energy and consumables costs of the membrane processes to DAF.

## 2. Results and Discussion

In the current work, five NF and five RO membranes were evaluated for polishing step purification of wastewaters generated in rendering facility following primary treatment by UF. Table 1 lists the RO and NF membranes tested, including their specifications: membrane type, material of construction, manufacturer, and pure water permeability. Membranes were selected to provide a range of manufacturer-reported characteristics, as there often is a trade-off between effluent quality and productivity. Measured pure water permeability reported in Table 1 was found to match closely with manufacturer-reported values.

### 2.1. Membrane Performance during Direct-Flow Filtration

Direct-flow filtration was used for membrane screening to compare performance for a variety of membranes. Membrane performance metrics of interest for direct-flow studies are flux— volume/area/time—*versus* volume processed and selectivity—high rejection of dissolved solids. In this study, UF pretreated wastewater was used as the feed stream.

**Figure 2:**
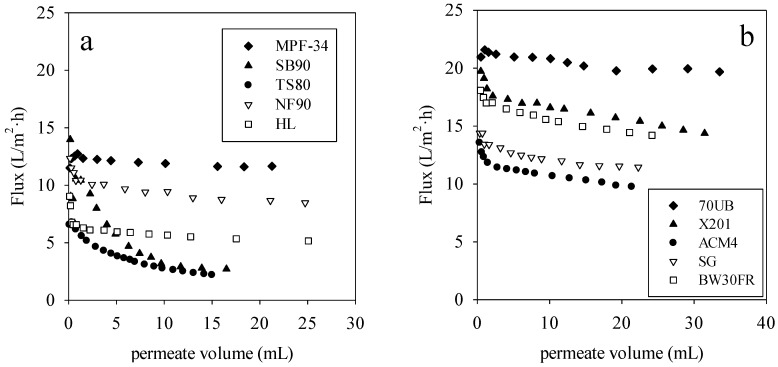
(**a**) Flux measurements by direct-flow filtration using NF membranes and (**b**) flux measurements by direct-flow filtration using RO membranes. Transmembrane pressure (TMP) was adjusted for each membrane to achieve a constant initial flux for all of the membranes. Using constant initial flux enables direct comparisons among membranes.

Figure 2a,b show membrane flux *versus* permeate volume for direct-flow filtration rendering facility wastewater by the NF and RO membranes. Table 2 provides the specific transmembrane pressure used for each membrane. These pressures were selected to provide the same starting flux based on pure water permeability data. For all these membranes, there was significant flux reduction from initial fluxes, which is attributed to the additional resistance induced by membrane fouling and concentration polarization. From Figure 2a, the MPF-34 membrane had the highest stable flux of any of the NF membranes. From Figure 2b, the 70UB membrane had the highest stable flux of any of the RO membranes.

**Figure 3:**
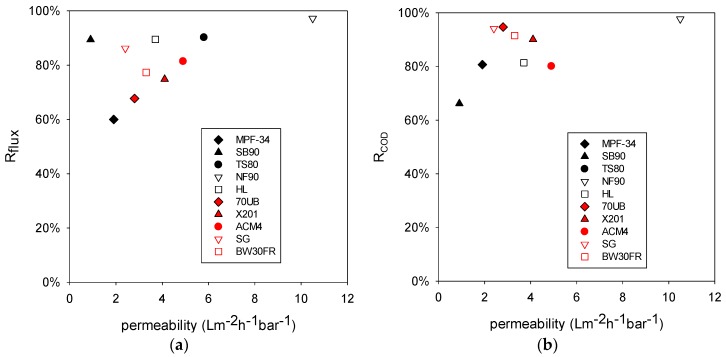
(**a**) Percentage flux reduction for RO and NF membranes and (**b**) percentage COD reduction.

Figure 3a illustrates the role that membrane permeability plays on reduction of flux. Percentage reduction of flux (R_flux_) was calculated using Equation (1). F_stb_ is the stable flux (Lm^−2^·h^−1^), and F_int_ is the initial flux (Lm^−2^·h^−1^).

(1) $$R_{\text{flux}}=\left(\frac{F_{\text{int}}-F_{\text{stb}}}{F_{\text{int}}}\right)\cdot 100\%$$

Figure 3a shows that R_flux_ does not correlate strongly with the membrane permeability. R_flux_ is associated with membrane fouling. The most permeable membrane NF90 did have the highest R_flux_, indicating that this membrane was more severely fouled than other membranes. However, in general membrane type (*i.e.*, NF or RO) does not seem to play a significant role here. Figure 3b compares percentage COD reduction to membrane permeability. COD reduction is defined in Equation 2. RO membranes (red symbols) had higher R_COD_ than NF membranes (black symbols) with similar permeability. As mentioned earlier, RO membranes have lower MWCO [4,5], which may be able to sieve larger organic molecules than the NF membranes, leading to higher R_COD_. The most severely fouled membrane NF90 had a high R_COD_ compared to other NF membranes. This finding may be associated with the foulant layer on the membrane surface. While this layer increased the transport resistance, it also improved the sieving capability and increased COD reduction.

(2) $$R_{\text{COD}}=\left(\frac{{\text{COD}}_{\text{feed}}-{\text{COD}}_{\text{filtrate}}}{{\text{COD}}_{\text{feed}}}\right)\times 100\%\;$$

Table 2 shows the characteristics of the UF pretreated wastewater samples used as a feed for the NF and RO membranes in the second polishing step, along with data from the permeate quality measurements. For all membranes, there were substantial reductions in COD and TDS. The turbidity was reduced by nearly 100% for all the membranes tested. In general, RO membranes provided higher salt rejection (*i.e.*, lower TDS) and rejection of compounds contributing to COD. Table 2 also summarizes the pseudo-steady-state permeability. RO membranes generally had higher pseudo-steady-state permeability than NF membranes, consistent with measured and reported pure water permeability. Permeability depends on porosity and nominal pore size. It therefore is possible that differences in these properties among the various membranes play a role in the measured permeability.

### 2.2. Membrane Performance during Cross-Flow Filtration

Based on the direct-flow productivity and selectivity, two membranes (MPF-34 and 70UB) were selected for further consideration in cross-flow studies. During cross-flow studies, we measured permeability *versus* volume processed for 96-h runs using wastewater that had been pretreated using UF. For the MPF-34 and 70UB membranes, a TMP of 14.5 bar and 22.4 bar, respectively, was used to maintain consistency with the direct-flow study.

**Figure 4:**
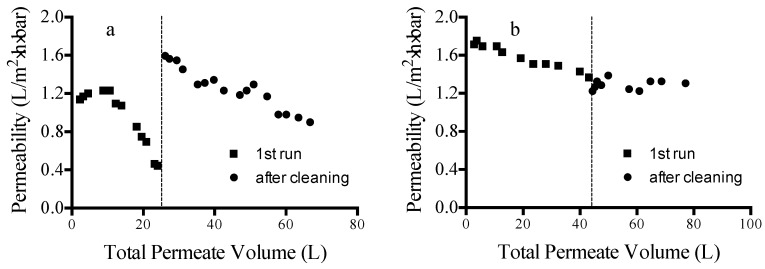
(**a**) Cross-flow filtration results for MPF-34 NF membrane. Each run was done for a period of 96 h. The vertical dashed line indicates the point where CIP was done with NaOH solution. (**b**) Cross-flow filtration results for 70UB RO membrane. Each run was done for a period of 96 h. The vertical dashed line indicates the point where CIP was done with SDS solution.

Figure 4 shows long-term (96 h) cross-flow filtration results for MPF-34 (Figure 4a) and 70UB (Figure 4b) membranes. Permeability data are given for runs with pristine membranes (1st run) and after cleaning. Two different cleaning protocols were examined. For MPF-34, cleaning was done using a caustic solution (aqueous NaOH). For 70UB, cleaning was done using a surfactant solution (aqueous sodium dodecyl sulfate, SDS). In both cases, CIP (cleaning in place) was done by flowing the cleaning solutions through the test cell for a period of 1 h.

Figure 4a shows that, despite its good performance in short-term direct-flow testing, the MPF-34 NF membrane fouls significantly during long-term cross-flow testing. Cleaning with caustic solution restores flux fully; however, the permeability again begins to decrease over time following the cleaning step. The reason for the higher permeability post-cleaning is not clear, but may be due to partial hydrolysis of the selective layer during exposure to the caustic solution. Figure 4b shows that the 70UB RO membrane effectively maintains permeability during long-term testing. Its capacity prior to cleaning is roughly 1600 L/m^2^. Unlike cleaning done with caustic solution, cleaning with a surfactant solution was not effective for restoring permeability to the initial value. The cleaning results indicate that fouling may be due to inorganic scaling rather than fouling by organics. Nevertheless, 70UB approaches steady-state operation following the initial cleaning step.

### 2.3. Membrane Fouling Characterization

SEM and EDS were used to visualize the morphology and fouling of membrane surfaces and to perform elemental analysis of the membrane surfaces before and after filtration.

**Figure 5:**
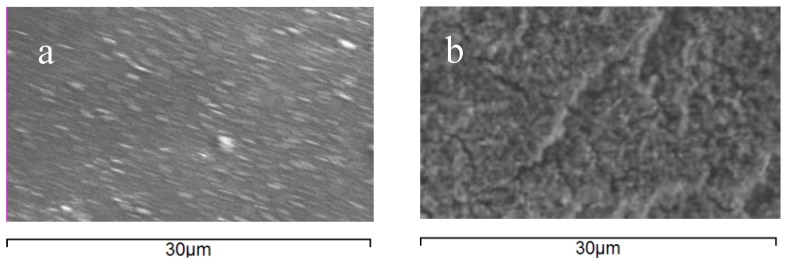
SEM images of MPF-34 membrane surface before (**a**) and after (**b**) filtration.

Figure 5 shows SEM images of the MPF-34 membrane surface before and after filtration. Similar results were obtained for the 70UB membrane. There is a visual change in the surface morphology of the membrane surface after fouling. Table 3 summarizes the elemental composition of the surface from EDS measurements. Before fouling, the surface comprises C, N, and O, characteristic of the amide chemistry used as the selective layer. After fouling, N is no longer observed, indicating two things: First, the foulant is not protein-based, and the base membrane is coated with a layer of foulant that is thicker than the penetration depth of the EDS measurement (<5 micron). Second, oxygen content increased dramatically. In addition, three new elements appear prominently in the spectrum: Fe, Zn, P. The foulants therefore appear to include inorganic oxide salts. This result is consistent with the results from cleaning, which showed that caustic was effective in restoring flux, whereas the surfactant solution used for organics removal was not.

**Figure 6:**
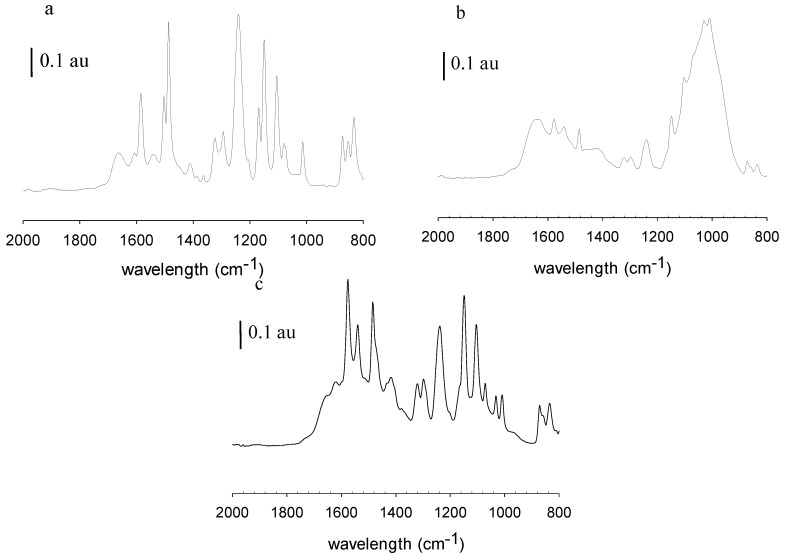
FTIR of (**a**) pristine MPF-34 membrane; (**b**) fouled MPF-34 membrane; (**c**) cleaned MPF-34 membrane.

**Figure 7:**
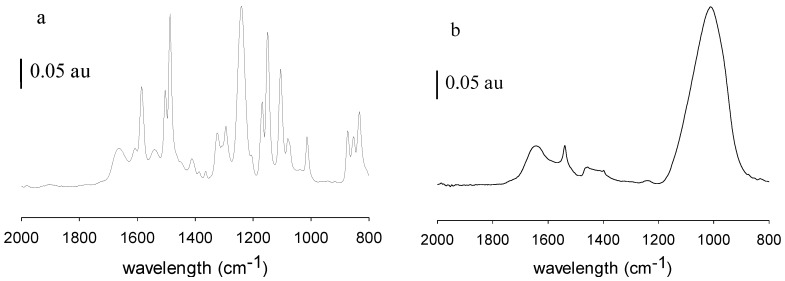
FTIR of (**a**) pristine 70UB membrane; (**b**) fouled 70UB membrane.

Figure 6 shows the ATR-FTIR spectra of the pristine, fouled and cleaned MPF-34 membrane. Spectrum a represents the pristine membrane. Spectrum b represents the membrane after filtration with rendering facility wastewater but before membrane cleaning. Spectrum c represents the membrane following filtration and membrane cleaning. Figure 7 shows the corresponding images for 70UB membranes. These results show significant changes in the chemical nature of the NF and RO membrane surfaces post-filtration, before membrane cleaning. ATR-FTIR is a surface-sensitive technique that provides details on the surface chemistry within the first 0.5–5 µm (depending on the wavenumber) of the sample. Peaks associated with the base membranes diminish in intensity as the membranes become fouled and may disappear entirely if the foulant layer thickness exceeds the IR light penetration depth. From Figure 6a and Figure 7a, MPF-34 and 70UB membranes show similar spectra. They both have sharp absorption bands at 1645 cm^−1^ and 1535 cm^−1^, assigned to the amide I and amide II bands of the polyamide material [9]. They also have absorption peaks at 1580 cm^−1^ and 1490 cm^−1^, assigned to polysulfone groups [10]. As indicated in Figure 6b and Figure 7b, on both fouled surfaces, these peaks diminished and a broad peak appeared centered at 1010 cm^−1^, which might be attributed to PO_4_ [11] or polysaccharides [9]. It suggests the membrane surface was covered by some organic layer in addition to the inorganic foulants, consistent with findings by EDS. Figure 6c is the cleaned MPF-34 surface. Again, it validates that caustic cleaning was effective in cleaning the membrane, as base membrane peaks reappear.

### 2.4. Operating Cost Analysis

Table 4 compares the estimated costs of energy and consumables for DAF to those for membrane treatment steps. Data are presented for total annual operating costs to run a 160 gal/min process. In this table, all membrane lifetimes were expected to be only 1 year, far shorter than the manufacturer‘s suggested lifetimes. Table 5 compares the costs using the suggested lifetimes of 2 years for UF membranes and 5 years for NF and RO membranes.

The cost data for the DAF process was provided by our industry partners. As shown in Equation (3), the total cost of the membrane process (C_tot_, $/year) comprises the cost of membrane consumption (C_membr_, $/year), the energy cost (C_energy_, $/year) associated with pumping, and the cleaning cost (C_clean_, $/year).

(3) $$C_{\text{tot}}=C_{\text{membr}}+C_{\text{energy}}+\;C_{\text{clean}}$$

Equation (4) shows that the membrane cost is determined by the membrane area required in the process, S_membr_ (m^2^); the membrane cost per unit area, C’_membr_ ($/m^2^), which was provided by the membrane suppliers; and the life time of the membrane, t_1_ (year), which was varied in Table 4 and Table 5.

(4) $$C_{\text{membr}}=\frac{C_{\text{membr}}^{\prime }\times S_{\text{membr}}}{t_{1}}$$

S_membr_ was estimated using Equation 5 by the total volume of wastewater to be treated per unit time, V_ww_ (L·h^−1^), and the membrane pseudo-steady-state flux, V_membr_ (Lm^2^·h^−1^). The flux values used in the calculation were based on the experimental work in this study and a previous study that evaluated the ultrafiltration step. Values for UF, NF and RO membranes were 9, 11, and 15 Lm^−2^·h^−1^ in this estimation.

(5) $$S_{\text{membr}}=\frac{V_{\text{ww}}}{V_{\text{membr}}}$$

C_energy_ was calculated using Equations (6) and (7), where C’_energy_ is the unit energy cost ($/kW), N_energy_ is the total energy consumption (kW), E (kWh) is the power of the pump required, and t is the operation time. In this study, cleaning was scheduled every 4 d and assumed to be 1 h long. The operation time is about 361.2 days/year.

(6) $$C_{\text{energy}}=C_{\text{energy}}^{\prime }\times N_{\text{energy}}$$

(7) $$N_{\text{energy}}=E\times t$$

Cleaning cost is usually 15%–20% of the operating cost [12]. It was assumed to be 15% in this case.

From Table 4, the estimated operating costs for the combined UF/NF or UF/RO dual-stage membrane filtration process are less than about 62% of the current DAF process. That is, we estimate that the operating cost for the membrane treatment process to be $2.0/1000 gal (UF/NF) or $1.9/1000 gal (UF/RO), compared to $3.20/1000 gal for DAF. Chemicals were the largest cost center for DAF, representing nearly 94% of the overall treatment cost, compared to less than 2% of the overall cost for membrane steps. Membranes were the largest cost center for the membrane process. In Table 5, a more realistic UF membrane lifetime of 2 years is assumed with a lifetime of 5 years for the NF or RO membranes. The cost estimates drop to $1.2 and $1.4 per 1000 gallons of wastewater, respectively, less than about 45% of the DAF process. Not surprisingly, anything that can extend membrane lifetime (e.g., reducing the frequency of cleaning by making the surface fouling-resistant) will have a direct economic benefit.

## 3. Materials and Methods

### 3.1. Materials

Commercial, M-series GE Septa™ cross-flow UltraFilic flat-sheet UF membranes with a nominal MWCO of 100 kDa were purchased from Sterlitech Company (Kent, PA, USA). Commercial flat-sheet RO and NF membranes were also purchased from Sterlitech Company. COD digestion vials (high range, 20–1500 mg/L) were purchased from Hach Company (Loveland, CO, USA). The vials contained mercuric sulfate, chromic acid, silver sulfate, sulfuric acid and deionized (DI) water. Sodium hydroxide (NaOH, anhydrous, >97%) was purchased from Alfa Aesar (Ward Hill, MA, USA).

### 3.2. Rendering Facility Wastewater

Polyacrylamide-free wastewater prior to DAF was collected from a local rendering facility (Ward, SC, USA). The wastewater was stored in plastic containers at ~2 °C until filtration. The wastewater contains a high concentration of proteins and fats. In their characterization of wastewater from this same facility, Wandera and Husson [1] found fluctuations in the composition of wastewater samples, which had COD ranging from 29,000 to 97,000 mg/L and total solids from 11,000 to 47,000 mg/L. This wastewater was pretreated by cross-flow filtration through commercial GE M-series UltraFilic UF membranes (shown as the first stage in Figure 1). To avoid fouling, M-series UltraFilic membranes are engineered to be extremely hydrophilic [13,14]. This pretreatment step removes many of the organic foulants that affect the tighter NF and RO membranes. The TDS level of the pretreated wastewater samples used as a feed for the NF and RO membranes was 4000–6800 mg/L. The COD level was 8300–8600 mg/L. The pH of the wastewater is about 5.8.

### 3.3. Membrane Filtration

#### 3.3.1. Direct-Flow Filtration

Direct-flow membrane filtration experiments were carried out using each of the NF and RO membranes listed in Table 1. Measurements were done using a Sterlitech HP4750 stirred cell (Sterlitech Company). The membrane cell system accommodates a 49-mm diameter membrane and presents an effective membrane test area of 14.6 cm^2^. For each membrane in Table 1, the reported pure water permeability was measured first and used to select TMP that would achieve a constant initial flux for all of the membranes. Using constant initial flux enables direct comparisons among membranes. The target flux values for the NF and RO membrane sets were set by using the highest recommended pressure for those NF and RO membranes that had the lowest pure water permeability. For the NF membranes, target initial flux used to calculate TMP for individual membranes was 40 L/m^2^·h. For the RO membranes, it was 63 L/m^2^·h. For performance comparisons using rendering facility wastewater, direct-flow filtration measurements were done using UF-treated wastewater as the feed. Permeate flux values were calculated from the mass of permeate collected over time.

#### 3.3.2. Cross-Flow Filtration

Cross-flow membrane filtration experiments were carried out using one NF (Koch MPF-34) and one RO (Toray 70UB) membrane that showed stable flux during direct-flow filtration experiments. UF-treated wastewater was used as the feed, and productivity and capacity of the membranes were measured. Measurements were done using a Septa^®^ CF II medium/high foulant membrane cell system (GE Osmonics, Minnetonka, MN, USA). The membrane cell system accommodates a 19 cm × 14 cm flat sheet membrane and presents an effective membrane test area of 140 cm^2^. The wastewater was circulated using a Hydra-Cell pump (Wanner Engineering, Inc., Minneapolis, MN, USA). Details of the feed container were presented earlier [1]. Experiments were carried out using a TMP, selected depending on the membrane that was being tested, as discussed in Section 3.3.1. Permeate flux values were calculated from the permeate volumes collected at different times.

Membrane cleaning to detach accumulated foulants was conducted after 96 h. Cleaning was done by rinsing the membrane surface at room temperature for 1 h with either a 1 wt % NaOH solution (pH 11.6) or 0.5 wt % aqueous SDS solution (pH 8.5). Filtration with rendering wastewater was repeated after the cleaning to determine the percentage recovery of the original permeate flux achieved by the membrane cleaning step and hence to evaluate the effectiveness of the cleaning method.

### 3.4. Membrane Physicochemical Characterization

#### 3.4.1. ATR-FTIR

ATR-FTIR spectroscopy was used to characterize surface chemical properties of the membranes before and after filtration, as well as membranes after filtration and cleaning. These measurements were done to detect membrane fouling and the degree to which cleaning removed organic foulants. Spectra were obtained using a Thermo-Nicolet Magna 550 FTIR spectrometer (Thermo Fisher Technologies Inc., Fair Lawn, NJ, USA) equipped with a diamond ATR accessory. Measurements were done according to a procedure detailed elsewhere [15].

#### 3.4.2. SEM and EDS

SEM and EDS were done to visualize the morphology of membrane surfaces and to perform elemental analysis of the membrane surfaces before and after filtration. Images were obtained using a Hitachi S-4800 SEM (Hitachi, Tokyo, Japan) equipped with an Oxford INCAx-act EDS (Oxford, UK). Representative 0.5 cm^2^ samples of the membranes were attached with carbon tape to aluminum stabs prior to the SEM measurements. The SEM measurements were performed at an accelerating voltage of 20 kV and magnifications of 2000× and 5000×.

#### 3.4.3. Water Quality Analysis

##### TDS

The TDS concentration of the feed and permeate were measured using a multiple parameter SympHony^TM^ meter (VWR International, LLC, Radnor, PA, USA). The meter was calibrated using standard solutions with known salt concentrations (catalog numbers 2236.10-32, 2244.50-32, 2241-32) purchased from Ricca Chemical Company (Arlington, TX, USA).

##### COD

The COD of the feed and permeate were measured using the closed-reflux, colorimetric method using dichromate ion as the oxidant. Water samples were diluted by a factor of up to 100 using DI water to ensure that the samples had COD within the detection range (20–1500 mg/L) of the digestion solution vials. All measurements were done in triplicate according to a procedure detailed elsewhere [1]. COD was determined from a calibration plot that was prepared using standardized COD solutions (catalog number 22539-29, Hach Company).

##### Turbidity

Turbidity was measured using a MICRO 100 Laboratory Turbidimeter (HF Scientific, Fort Myers, FL, USA). This turbidimeter measures and records the turbidity of a sample in nephelometric turbidity units (NTU) and has a measurement range of 0–1000 NTU. The meter was calibrated using a calibration kit containing TOC standards (catalog number 39957, HF Scientific).

## 4. Conclusions

In this project, we studied the performance of commercial NF and RO membranes using rendering facility wastewater samples following initial purification by an additive-free UF step. Because the influent to a rendering facility wastewater treatment system can change hourly, daily, or weekly depending on what is processed, it is not possible to draw generalized conclusions about which membrane or membrane type works most effectively for treatment of all rendering facility wastewaters. However, we can conclude that direct-flow filtration provides an effective and efficient screening tool to reduce the level of effort in long-term cross-flow measurements. It should be used as a tool to down-select membranes for each new high-strength wastewater application.

Preliminary cost analysis compared the costs of energy and consumables for DAF and a two-stage UF/NF or UF/RO filtration process. However, a detailed cost analysis should be undertaken for the dual-stage membrane filtration process. That is to say, the numbers provided in this work are preliminary estimates and need more scrutiny.

Membrane cleaning was not an objective of this project; however, two preliminary experiments were done. While we cannot generalize the findings to wastewaters from other facilities, we can conclude that the use of elemental mapping on fouled membrane surfaces provides clues on the nature of the foulant that can be used to guide cleaning studies.

## Figures and Tables

**Table 1:** Nanofiltration and reverse osmosis membranes used in the study.

Membrane Product	Type	Material	Manufacturer	Pure Water Permeability (Lm^−2^·h^−1^·bar^−1^)
MPF-34	NF	Proprietary	Koch Membrane	1.9
SB90	NF	Cellulose Acetate Blend	TriSep	0.9
TS80	NF	Polyamide	TriSep	5.8
NF90	NF	Polyamide	Dow	10.5 ^1^
HL	NF	Proprietary	GE Osmonics	3.7
70UB	RO	Polyamide	Toray	2.8
X201	RO	Polyamide-urea	TriSep	4.1
ACM4	RO	Polyamide	TriSep	4.9
SG	RO	Proprietary	GE Osmonics	2.4
BW30FR	RO	Polyamide	Dow	3.3

^1^ Permeability for this membrane was calculated based on data provided by the supplier.

**Table 2:** Performance data for nanofiltration and reverse osmosis membranes used in the study.

Parameter	MPF-34 1	SB90 1	TS80 1	NF90 2	HL 2	70UB 1	X201 1	ACM4 1	SG 1	BW30FR 1
TDS Reduction	74%	61%	22%	98%	N/A	90%	91%	80%	90%	70%
COD Reduction	81%	66%	24%	98%	81%	95%	90%	80%	94%	92%
Steady State Permeability (LMH/bar)	0.76	0.10	0.57	0.29	0.39	0.90	1.03	0.91	0.33	0.75
TMP (bar)	15.8	31.5	5.3	31.1	15.5	22.1	15.5	11.0	35.9	20.0

^1^ Feed TDS level was 4060 mg/L and COD was 8300 mg/L. ^2^ Feed TDS level was 6800 mg/L and COD was 8600 mg/L.

**Table 3:** Elemental composition of the membrane surfaces from EDS measurements.

Element	Atomic Percentage	
	Pristine	Fouled
C	52.2	28.4
O	22.9	49.5
N	24.9	-
P	–	7.5
Fe	–	11.1
Zn	–	3.5

**Table 4:** Annual cost estimations of membrane operations based on our lab scale rendering facility wastewater treatment data. In this calculation, membrane lifetimes are assumed to be only 1 year.

Unit Operations	Pumping Cost ^2^	Membrane/Chemical Cost ^3^	Annual Total Operating Cost	Cost % Compared to DAF
Ultrafiltration ^1^	$17,300	$31,900	$56,600	21
Nanofiltration ^1^	$44,400	$52,200	$111,100	41
Reverse Osmosis ^1^	$62,100	$29,000	$104,800	39
DAF	$8,400	$260,700	$269,100	–

^1^ UF data are based on operation under 6.5 bar working pressure, while NF and RO data are based on operation under 14.5 bar and 22.4 bar, respectively. ^2^ The pump is 13.9, 35.6, and 49.8 hp for UF, NF and RO membranes, respectively. The pump efficiency is assumed to be 60%, and the capacity is 263 gal/min for all three membrane processes. ^3^ Membrane cost was considered in the membrane process, while chemical usage cost was considered in the DAF process.

**Table 5:** Annual cost estimations of membrane operations based on our lab scale rendering facility wastewater treatment data. In this calculation, manufacturer suggested membrane lifetimes are assumed.

Unit Operations	Pumping Cost ^2^	Membrane/Chemical Cost ^3^	Annual Total Operating Cost	Cost % Compared to DAF
Ultrafiltration ^1^	$17,300	$16,000	$38,300	14
Nanofiltration ^1^	$44,400	$10,400	$63,100	23
Reverse Osmosis ^1^	$62,100	$5,800	$78,100	29
DAF	$8,400	$260,700	$269,100	–

^1^ UF data are based on operation under 6.5 bar working pressure, while NF and RO data are based on operation under 14.5 bar and 22.4 bar, respectively. ^2^ The pump is 13.9, 35.6, and 49.8 hp for UF, NF and RO membranes, respectively. The pump efficiency is assumed to be 60%, and the capacity is 263 gal/min for all three membrane processes. ^3^ Membrane cost was considered in the membrane process, while chemical usage cost was considered in the DAF process.

## Abbreviations

The following abbreviations are used in this manuscript:

ATR-FTIR	Attenuated total reflectance-Fourier-transform infrared spectroscopy
CIP	Cleaning in place
COD	Chemical oxygen demand
DAF	Dissolved air flotation
DI	Deionized
EDS	Energy dispersive X-ray spectroscopy
F_int_	Initial flux
F_stb_	Stable flux
MWCO	Molecular weight cutoff
NF	Nanofiltration
NTU	Nephelometric turbidity units
R_COD_	Reduction of chemical oxygen demand (%)
R_flux_	Reduction of flux (%)
RO	Reverse osmosis
SEM	Scanning electron microscopy
SDS	Sodium dodecylsulfate
TDS	Total dissolved solids
TMP	Transmembrane pressure
UF	Ultrafiltration
